# Genetic and environmental influences on early-age susceptibility and initiation of nicotine-containing product use: A twin-pairs study

**DOI:** 10.18332/tpc/173556

**Published:** 2023-11-21

**Authors:** Andrew Kochvar, Yadi Liu, Marcus Munafo, Zheng Xu, Hongying Daisy Dai

**Affiliations:** 1 College of Public Health, University of Nebraska Medical Center, Omaha, United States; 2 College of Osteopathic Medicine, Kansas City University, Missouri, United States; 3 Bristol Population Health Science Institute, University of Bristol, Bristol, United Kingdom; 4 Department of Mathematics and Statistics, Wright State University, Dayton, United States

**Keywords:** nicotine-containing product use initiation, nicotine-containing product use susceptibility, heritability, genetic factors, environmental factors, ABCD study

## Abstract

**INTRODUCTION:**

Nicotine-containing products (NCPs) such as electronic nicotine delivery systems (ENDS) are increasingly common throughout the landscape of youth use of nicotine-containing products (NCP), and have overtaken traditional cigarette smoking modalities. This study seeks to examine the genetic and environmental influences on liability for susceptibility and initiation of ENDS and other NCPs among US children.

**METHODS:**

Data were drawn from 886 monozygotic (MZ) and dizygotic (DZ) twin pairs aged 9–10 years in the Adolescent Brain & Cognitive Development (ABCD) study at the baseline during 2016–2018. Heritability (h^2^) measured the proportion of the total phenotypic variation attributable to genes. Variance component models were utilized to analyze influences from the common environment (c^2^) and unique environmental factors (e^2^), taking into account correlations within twin pairs.

**RESULTS:**

The national sample included 50% females, 69.5% of non-Hispanic Whites, 12.8% of non-Hispanic Blacks, and 11.6% of Hispanics, with a mean age of 121.5 months. The twin sets were 60% DZ and 40% MZ. Heritability was low for NCP susceptibility (h^2^=0) and moderate for NCP initiation (h^2^=39%, p=0.02). The variance associated with NCP susceptibility was primarily influenced by environmental factors, especially one’s unique factors (c^2^=37%, p<0.0001 vs e^2^=63%, p<0.0001). In contrast, the variance associated with NCP initiation was split across common and unique environmental factors (c^2^=32%, p=0.02 vs e^2^=29%, p=0.02).

**CONCLUSIONS:**

In the era with ENDS use surging among youth, NCP initiation remains to be a heritable trait with joint influence from the environment. NCP susceptibility is largely influenced by environmental factors, especially unique environments. Continued assessment of gene × environment interaction can better inform future youth NCP interventions.

## INTRODUCTION

The use of nicotine-containing products (NCPs) among youth has made a notable resurgence since the reversal of the downtrend in cigarette smoking. This is largely attributed to the rise in popularity of Electronic Nicotine Delivery Systems (ENDS) in school-aged children; these products now reign as the most common modality of NCP use by a significant margin^1^. Compared to traditional cigarettes, e-cigarettes utilize aromatic, sweet, or other food-related flavorings to a greater extent, which disproportionately draws young people to use these products^2^. Harm perceptions surrounding such products are low in youth populations due to media and advertisement portrayals as discrete and odorless alternatives to traditional combustible cigarettes^3^.

The health implications associated with early NCP use are well established. Nicotine is highly addictive and exposure to nicotine during adolescence can harm the developing brain during an important period of cognitive development^4,5^. Studies have shown that tobacco use during adolescence can negatively impact learning, memory, and attention^6,7^. ENDS aerosol contains a number of potentially toxic substances (e.g. carbonyl compounds and heavy metals)^5^; vaping is associated with increased risk of respiratory symptoms^8^, cardiovascular diseases^9^, and other adverse health outcomes^5^. No matter the modality of tobacco product use, it is not safe to use any tobacco products at a young age^1^.

National surveys have been pivotal in describing adolescent use of ENDS and other NCP products, including data surrounding patterns of NCP use. In 2021, 35.2% of US high school students (5.22 million in grades 9–12, typically aged 15–18 years) and 11.1% of middle school students (1.34 million in grades 6–8, typically aged 12–14 years) reported ever having used any NCP product, and the most common among those used between middle and high school users were e-cigarettes (5.3 million)^1^. The prevalence of adolescent NCP use tends to be higher in sexual minorities (vs heterosexual individuals)^10^ and those reporting psychological distress (vs not)^1^. However, genetics also play an important role in impacting a wide range of behavioral and health outcomes in children, including substance use. It is estimated that genetic influences of smoking initiation range 37–84% in women and 28–84% in men^11^. Youth behaviors are also shaped by a combination of individual and environmental factors, as somewhere between 40–60% of the variance for substance use disorder can be accounted for by the shared and non-shared environments^12^. To develop effective public health interventions, we need to better understand how genes and environment affect youth NCP use, especially at an early age. Behavioral genetic analysis that leverages the twin data can be utilized to characterize such influences between related individuals to delineate between environmental versus genetic effects^13,14^.

Existing cigarette use studies suggest that both genetic factors and shared environmental influences predict combustible cigarette initiation in child, adolescent, and adult populations^15,16^. Preliminary evidence from analysis of twins aged 10–15 years purported additive genetic and shared environmental influences are similarly responsible for both combustible cigarette and ENDS use, though ENDS initiation uniquely demonstrated individual environmental influences that differed from the combustible cigarette group^17^. A separate behavioral genetic analysis of a small sample of female twin pairs aged 16–21 years suggests shared environmental influences are predominantly responsible liability of ENDS initiation^18^. Susceptibility to cigarette use among adolescents is a validated measure in the prediction of future cigarette smoking initiation and regular use^19^. Prior studies have found that youth susceptibility to NCP use overlaps widely across different products^20^. A number of risky behaviors and environmental factors, such as the level of parental monitoring and school environment, also predict NCP use susceptibility^21^. However, it remains unclear about the effects of both genes and environment on NCP susceptibility and initiation, especially in the era of surging ENDS use among youth.

This study aims to understand and explain the extent to which additive genetic and environmental influences affect susceptibility to NCP use and liability for NCP initiation at an early age. To do so, we evaluated and compared monozygotic (MZ, ‘identical twins’) and dizygotic (DZ, ‘fraternal twins’) twins using data from the Adolescent Brain and Cognitive Development (ABCD) Study. This study explores three questions among children aged 9–10 years in 2016–2018, as e-cigarettes had become the most commonly used NCP: 1) ‘Do genetic and environmental factors influence youth early-age NCP use behaviors?’, 2) ‘Do identical pairs have a higher correlation in NCP use behaviors than fraternal pairs?’, and 3) ‘Does the hereditability vary between NCP use susceptibility and initiation?’. Evaluating genetic and environmental influences on this age-group can better inform public health practices to curb NCP use during an important period of physical and cognitive development.

## METHODS

The Adolescent Brain and Cognitive Development (ABCD) study is the largest and most comprehensive longitudinal assessment of brain development as well as child and adolescent health in the United States. Baseline participants aged 9–10 years were recruited across 21 US research sites between 1 October 2016 and 31 October 2018 through a probability sample of schools selected for sex at birth, race/ethnicity, socioeconomic status, and urbanicity. The weight variable provided in ABCD was generated using a propensity model of age, sex, and race/ethnicity and missing data imputation to ensure that weighted ABCD data maintain the sample demographics in accordance with the American Community Survey 3rd- and 4th-grade enrollment statistics at each site^22^. Participants are asked for in-person assessment sessions once a year for behavioral and biospecimen collections, with brief remote assessments (e.g. youth substance exposure/use) at 6 months between in-person sessions. All parents or guardians provided written informed consent, and children gave written assent. Study procedures were approved by the UC San Diego Central Institutional Review Board (IRB) and each local institutional IRB. This article followed the Strengthening the Reporting of Observational Studies in Epidemiology (STROBE) guideline for cohort studies^23^.

Data from this study were collected from the ABCD Twin Hub, a sub-study of monozygotic (MZ) and dizygotic (DZ) same-sex twins from four sites in Minnesota, Colorado, Virginia, and Missouri^14^. An equal number of twins born during 2006–2008 were recruited from registries of leading twin research centers at each site during 2016–2018. The twin dataset was accessed via the National Data Archive (NDA), with the removal of all personally identifiable information to safeguard the confidentiality and anonymity of the participants.

### Measures

*Zygosity*

Parents reported the relationship of the participants in his or her family with response options ‘Single’, ‘Sibling’, ‘Twin’, and ‘Triplet’. For those identified as twins, the genetically inferred zygosity status was first used to classify participants as MZ versus DZ twins. For those with missing zygosity status, the similarity of physical attributes rated by researchers was used to differentiate between MZ versus DZ twins, and parameters included facial appearance, complexion, hair color, hair texture, hair curliness, hair pattern, amount of hair, ear appearance, hair darkness, hair type, and eye color^14^.

**Figure 1:**
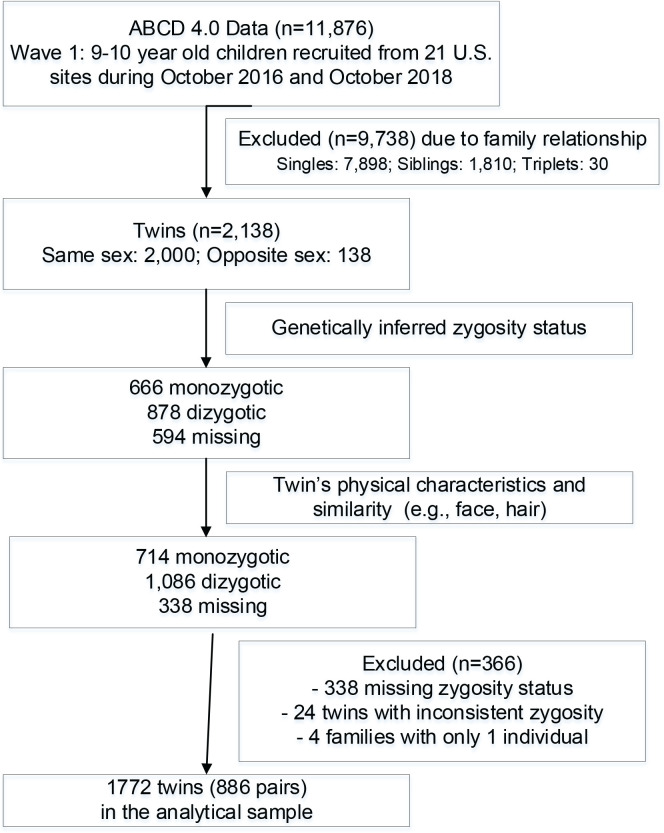
Flowchart for participants included in the twin pairs study analytical sample, Baseline ABCD Study, 2016–2018

Twin status was estimated from the ABCD dataset to better characterize the study population as demonstrated in Figure 1. Of 11876 children enrolled in the ABCD study at baseline, we excluded 9783 participants based on their family relationships (i.e. 7898 singles, 1810 siblings, and 30 triplets), resulting in 2138 self-reported twin children. We further excluded 366 children based on their genetically inferred zygosity status and twin’s physical characteristics and similarities. Ultimately, 886 pairs of twins (1732 participants) were utilized for the present study.

*Susceptibility to NCP use*

Participants were first asked whether they had heard of tobacco products, such as cigarettes, smokeless tobacco, cigars, hookah, electronic or e-cigarettes. Those who reported ‘Yes’ were asked whether they had ever tried any tobacco products. Those who reported ‘No’ were classified as never NCP users and were asked three separate questions: ‘Have you ever been curious about using a tobacco product such as cigarettes, e-cigarettes, hookah, or cigars?’, ‘Do you think you will try a tobacco product soon?’ and ‘If one of your best friends were to offer you a tobacco product, would you try it?’. Those who responded with an absence of firm decision (i.e. ‘Very curious’, ‘Somewhat curious’, ‘A little curious’ for the first question and ‘Definitely yes’, ‘Probably yes’, ‘Probably not’ for the latter two questions) were classified as ‘susceptible to NCP use’^21^.

*NCP Initiation*

In the timeline flow-back survey, participants were first asked whether they had heard of tobacco products, such as cigarettes, smokeless tobacco, cigars, hookah, electronic or e-cigarettes. Those who reported ‘Yes’ were further asked whether they have ever tried any tobacco products in their life by separate questions for each type of product (i.e. e-cigarette, cigarette, cigar, smokeless tobacco, hookah, pipe, and nicotine replacement). Those who responded ‘Yes’ were classified as ever NCP users^4^.

### Parent-reported sociodemographic factors

Sociodemographic factors to describe sample characteristics included age (months), sex at birth (male/female), race/ethnicity (non-Hispanic White, Non-Hispanic Black, Hispanic, and other Non-Hispanic races), parents’ education level (less than high school, high school, some college or associate degree, Bachelor’s degree, or postgraduate degree), family income ($) (<25000, 25000–49999, 50000–74999, 75000–99999, >100000, or don’t know/refuse to answer), the experience of family difficulty in the past 12 months (Yes/No), and whether the child was born prematurely (Yes/No). Measures on perceived neighborhood safety and crime were reported separately from youth and parents with higher scores indicating a safer view of the neighborhood^24^. Related survey questions are provided in Supplementary file Table 1.

### Statistical methods

Weighted descriptive statistics are reported for the overall sample and stratified by zygosity. Rao-Scott chi-squared tests were performed to detect group differences. Variance component models were performed to dissect the genetic and environmental influences associated with NCP use susceptibility and initiation. ACE mixed model analysis accounting for correlations within twin pairs was constructed to characterize phenotypic variability across additive genetic (A), shared environmental (C), and unique environmental (E) contributions in MZ and DZ twin pairs^17^. Additive genetic influences consider the effect of alleles on the variable of interest. Shared environmental influences are defined as experiences and exposures that remain constant within the twin-pair environment (e.g. family dynamics), while unique environmental effects consider mutually exclusive environmental influences not shared between twin pairs (e.g. peer influences). The latent sources of variance were estimated from differences in magnitude between correlation coefficients in evaluating twin pairs between conditions using phenotypic comparisons^25^. Differences in the magnitude of correlation between zygosity-based phenotypes therefore are attributable to the hypothesized source of variance, which must be either additive genetic or shared environmental. After testing ACE models, AE models or CE models were performed by removing the insignificant component.

Since MZ twins share the same genes and DZ twins share an average 50% of segregating genes, we expect a higher correlation in MZ twins than in DZ twins for an inheritable trait. Thus, the within-pair covariance of DZ twins was set to be half of that for MZ twins^26^. Heritability (h^2^), and percentage of variances from shared (c^2^) and unique (e^2^) environments are reported alongside Pearson correlation coefficients and Bayesian information criterion (BIC) to inform the model fit. Statistical analyses were performed using SAS with a 95% confidence level (p<0.05).

## RESULTS

The final analytical sample comprised 886 twin pairs with mean age of 121.5 months (standard error, SE=0.17). The sample had an equal male–female distribution, and twin sets were 60% DZ and 40% MZ (Table 1). The sample included a diverse population with 69.5% Whites, 12.8% Blacks, and 11.6% Hispanics. MZ and DZ twins had comparable socioeconomic status by sex, race/ethnicity, family income and neighborhood perceptions. The discrepancies in other sociodemographic categories (e.g. parental education level, family difficulty, and being premature) were generally small, with MZ twins more likely to be from low-income families, born prematurely, and slightly older than DZ twins.

**Table 1:** Participant characteristics of the baseline ABCD study, 2016–2018 (N=886 pairs)^a^

*Characteristics*	*Overall*	*Twin status*		
	*n (%)*	*Dizygotic n (%)*	*Monozygotic n (%)*	*p^b^*
**Total**	1772 (100)	1070 (59.9)	702 (40.1)	
**Age** (months), mean (SE)	121.5 (0.17)	121 (0.22)	122.3 (0.27)	0.0002
**Sex**				0.4035
Male	905 (50.0)	537 (49.1)	368 (51.4)	
Female	867 (50.0)	533 (50.9)	334 (48.6)	
**Race/ethnicity**				
White	1159 (69.5)	696 (69.2)	463 (70)	
Black	246 (12.8)	158 (13.5)	88 (11.6)	0.5854
Hispanic	192 (11.6)	118 (11.6)	74 (11.6)	
Other	174 (6.2)	97 (5.7)	77 (6.9)	
**Parental education level**				
High school or lower	43 (3.2)	33 (4.1)	10 (2.0)	
High school diploma	109 (7.6)	67 (7.7)	42 (7.5)	
Some college or associate degree	436 (28.8)	232 (25)	204 (34.4)	0.002
Bachelor’s degree	618 (32.7)	382 (34.2)	236 (30.4)	
Postgraduate degree	566 (27.7)	356 (29)	210 (25.8)	
**Family income** ($)				
<25000	126 (9.6)	90 (10.7)	36 (8)	
25000–49999	160 (13.3)	86 (11.9)	74 (15.4)	
50000–74999	240 (18.4)	146 (18.4)	94 (18.4)	0.2411
75000–99999	251 (14.1)	151 (14.7)	100 (13.0)	
≥100000	893 (38.8)	543 (39)	350 (35.5)	
Don’t know or refuse to answer	102 (5.8)	54 (5.3)	48 (2.6)	
**Family difficulty^c^**				
No	1596 (87.1)	972 (89.2)	624 (84.1)	0.01
Yes	176 (12.9)	98 (10.8)	78 (15.9)	
**Premature**				
No	763 (42.9)	499 (46.1)	264 (38.2)	0.0034
Yes	994 (57.1)	559 (53.9)	435 (61.8)	
**Neighborhood perceptions^d^**, mean (SE)				
Child	4.1 (0.03)	4.1 (0.04)	4.1 (0.04)	0.9801
Parent	4.2 (0.02)	4.1 (0.03)	4.2 (0.04)	0.0562

a Weighted descriptive statistics of participant characteristics are reported, overall and stratified by zygosity.

b Rao-Scott chi-squared tests were performed to detect group differences. Individual subject’s inverse probability weighting score was included as sampling weight to account for non-responsiveness and ensure population-valid estimates.

c Experience of any family difficulty in the past 12 months were assessed by 7 items, e.g. ‘need food but couldn’t afford it’, ‘didn’t pay the full amount of the rent or mortgage because you could not afford it’ (Cronbach’s alpha=0.91).

d Perceived neighborhood safety and crime assessed feelings about safety and the presence of crime in the respondent’s neighborhood, including measures from the youth (one item with a 5-point Likert scale, ‘My neighborhood is safe from crime’) as well as the parents (average of three items with a 5-point Likert scale: ‘I feel safe walking in my neighborhood, day or night’, ‘Violence is not a problem in my neighborhood’, and ‘My neighborhood is safe from crime’. Cronbach’s alpha=0.89). Higher scores indicated a safer perceived neighborhood (1=strongly disagree to 5=strongly agree). SE: standard error.

Overall, 18.8% of youth reported susceptibility to NCP use, including 19.1% for DZ individuals and 18.4% for MZ individuals. About 1.8% of twins reported NCP initiation, including 1.7% for DZ and 1.9% for MZ individuals.

Table 2 presents results from our modeled comparisons of variance between heritability (h^2^), common environmental (c^2^), and individual environment (e^2^) using the optimized ACE model and post hoc CE comparisons. The correlations of NCP susceptibility within DZ (r=0.21, p<0.0001) and MZ twin (r=0.19, p=0.002) pairs were found to be roughly equal, therefore, variance is heavily attributed to the environment. ACE model analysis further demonstrated the dominant role of unique environmental factors (e^2^=63%, p<0.0001), followed by the common environment to a less extent (c^2^=37%, p<0.0001). Additive genes do not seem to influence variance (h^2^=0%) of NCP susceptibility.

**Table 2:** Variance component analysis for NCP related traits in early childhood of baseline ABCD Study, 2016–2018 (N=886 pairs)

	*NCP susceptibility*	*NCP initiation*
**Prevalence** (% yes)		
Overall	18.8	1.8
Dizygotic individuals	19.1	1.7
Monozygotic individuals	18.4	1.9
**Pearson correlation**		
Dizygotic twins	0.21 p<0.001	0.13 p=0.002
Monozygotic twins	0.19 p=0.002	0.44 p<0.001
**ACE model^a^**		
h^2^ (heritability from additive genes)	0	0.39 (0.07–0.70) p=0.02
c^2^ (common environment)	0.37 (0.24–0.51) p<0.0001	0.32 (0.04–0.59) p=0.02
e^2^ (unique environment)	0.63 (0.49–0.76) p<0.0001	0.29 (0.05–0.54) p=0.02
BIC	1448.0	252.7

a Dominant genetic components (D) were excluded from the analysis to avoid non-identifiability and model overfitting. Variance component methods (e.g. structural equation models) were performed using SAS Proc Mixed and Proc NLMixed. Multiple variance decompositions methods, including ADCE, ACE, DCE, AE, CE, etc., were compared using BIC, model goodness-of-fit statistics. The optimal models with the smallest BIC were selected and presented in the table.

MZ correlation coefficient (r=0.44, p<0.0001) was more than double that of DZ (r=0.13, p=0.002) for NCP initiation, suggesting that variance for NCP initiation is largely explained by genetic factors. ACE analysis reported additive genes as the largest source of variance (h^2^=39%, p=0.02), though common (c^2^=32%, p=0.02) and unique (h^2^=29%, p=0.02) environments also influence NCP initiation.

## DISCUSSION

This study sought to explain the effects of genes and environment on NCP susceptibility and initiation at a time when ENDS use in young populations has become the primary driver of NCP use. Within our diverse, nationally representative sample of children aged 9–10 years, the present behavioral genetic analysis between twin pairs suggests that differences in NCP susceptibility is attributable to environmental factors, and in particular unique environmental factors. Shared environment contributed to variance as well, though to a less extent. NCP susceptibility did not appear to be affected by additive genetic influences. Conversely, additive genetic factors demonstrated the strongest influence on observed variance for NCP initiation, though both unique and shared environments both explained variance in initiation as well.

Additive genetic sources of variance are heritable but not necessarily attributable to a single gene. Environmental sources of variance occur as either shared or individual factors that may influence NCP susceptibility and initiation. Examples include shared home dynamics, school and neighborhood environment, and NCP control policy at the local level. Unique environmental influences can also independently inform behavioral variation as it pertains to individual characterization (e.g. peer influence or marketing exposure) that are distinct within twin pairs, regardless of zygosity. The rise in popularity of ENDS use is a major contributor to the resurgence of NCP use among young people, as e-cigarette has surpassed cigarettes and has become the most prevalent NCP product used by US youths since 2014^27^. Our study presents results from one of the largest twin datasets to inform genetic predisposition and environmental risk of NCP use in late childhood.

Recent studies attest that shared environmental factors explain NCP initiation, whereas regular NCP use is best explained by heritable traits^18,28^. Our study adds to the literature that NCP susceptibility at an early age could be largely affected by unique environmental factors, while genes and environment both contribute to youth NCP initiation. The importance of environmental factors on NCP use susceptibility was also identified in our prior hierarchical study, which elucidated that internalizing problems could pose an increased risk of NCP use susceptibility, while parental monitoring and school environment could serve as shared environmental factors to reduce youth NCP susceptibility^21^.

Of note, prior literature describes a dynamic continuum whereby genetics influence NCP-seeking behavior as age increases through adolescence, whereas shared environment shapes behavior in late childhood and early adolescence^29,30^. Within this realm, genetic modulation by nicotine has demonstrated dose-dependent increases in nicotine use, dependence, and nicotinic acetylcholine receptor expression in adults, which lends to the postulation that earlier ENDS and other NCP initiation may increase the likelihood of becoming an addicted daily NCP user^7,31,32^, especially for those with existing behavioral genetic risk factors present.

Our analysis provides adequate power and representation for a more nuanced discussion of the behavioral genetics of NCP use in this particular age group. Unlike prior literature^18,28^, a unique environment is shown to profoundly influence susceptibility to NCP product use in this age group. This difference may be explained by our relatively young study population compared to other studies. Those who have experienced early life stressors are at higher risk for NCP initiation^33^. The e-cigarette industry might entice youth who are at low risk of initiating cigarettes or other substances to try ENDS. Many of these marketing strategies use bright colors, appealing flavors, and celebrity endorsements, which can make the product seem sleek and increase curiosity among people, particularly at a young age^3,34^. Additionally, we have explored the full scope of nicotine product use in a national sample, which reflects the significant and ever-increasing burden of ENDS use. Collectively, these results emphasize the need for age and developmentally appropriate public health interventions with a particular focus on high-risk subpopulations.

### Limitations

The findings of this study should be evaluated in light of the following limitations. First, children are prone to response bias when asked about NCP subjects such as nicotine product use due to social desirability bias, though test-retest reliability for self-reported NCP use in adolescents is strong^35^. Given this, these data may be under-representative of the true burden of use. Second, this study did not characterize the specific types of environmental influences beyond unique and shared ones, especially those that are known to increase the future risk of substance use. Future work would best capture more specific environmental factors including but not limited to exposure to individual or familial trauma, chaos in the household, food or housing insecurity, and social support versus isolation. Third, the present study captures a singular timepoint within an ongoing longitudinal study, which limits the predictive value of trends in the relative influences of behavioral genetic components on nicotine product use throughout adolescence. In addition, given the relatively small sample size of NCP use at baseline, we were unable to differentiate the type of NCP. Future studies should assess whether genetic and environmental factors differ by each type of NCP, especially between ENDS and combustible NCP products. Finally, rural families are underrepresented in the ABCD study population, though the study is designed to minimize selection bias and be epidemiologically informed enough to capture national representation and adequate diversity^36^.

## CONCLUSIONS

This study analyzed a large twin dataset and found that early-age NCP initiation remained to be a heritable trait while NCP susceptibility was largely influenced by environmental traits. Our findings provided evidence to inform future interventions in preventing and reducing adolescent e-cigarette and other NCP use. Additional behavioral genetic analyses are needed to understand the genetic and environmental influences on tobacco use behaviors, especially regular tobacco use as these children age. As mentioned above, these will help to inform both developmentally appropriate and age-appropriate public health interventions targeted to individuals with risk factors.

## Data Availability

This is a secondary data analysis of the ABCD study, which can be accessed via the National Data Archive (NDA).
