# Supplementary material for: Genetic and environmental influences on early-age susceptibility and initiation of nicotine-containing product use: A twin-pairs study
Source: Tob Prev Cessat. 2023 Nov 21;9:34. doi: 10.18332/tpc/173556 (PMC10660284; doi:10.18332/tpc/173556)
Supplement: Supplementary file 1 [file TPC-9-34-s1.pdf]

Supplementary file

Table 1. Examples of Related Survey Questionnaire in Baseline ABCD Study, 2016-2018

Measures	Survey Questions	Response options
NCP ever use	Have you heard of tobacco products, such as cigarettes, smokeless tobacco, cigars, hookah, electronic or e-cigarettes?	Yes/no
	Have you ever tried any tobacco products in your life? with separate questions for cigarettes, smokeless tobacco, cigars, hookah, electronic or e-cigarettes.	Yes/no
Susceptibility	Have you ever been curious about using a tobacco product such as cigarettes, e-cigarettes, hookah, or cigars?	"Very curious," "Somewhat curious," "A little curious," and "Not at all curious."
	Do you think you will try a tobacco product soon?	"Definitely yes," "Probably yes," "Probably not," and "Definitely not."
	If one of your best friends were to offer you a tobacco product, would you try it?	"Definitely yes," "Probably yes," "Probably not," and "Definitely not."
Socio-demographic factors	Was the child born prematurely?	Yes/No
	In the past 12 months, has there been a time when you and your immediate family experienced any of the following because you could not afford it?<br><br>Needed food<br>Were without telephone service	Yes/No

	Didn't pay the full amount of the rent or mortgage<br>Were evicted from your home for not paying the rent or mortgage?<br>Had services turned off by the gas or electric company, or the oil company wouldn't deliver oil<br>Had someone who needed to see a doctor or go to the hospital but didn't go<br>Had someone who needed a dentist but couldn't go	
Family relationship	Relationship of the participant in his or her family	0 = single; 1 = sibling; 2 = twin; 3 = triplet
Neighborhood safety and crime for children	My neighborhood is safe from crime.	1 = Strongly Disagree; 2 = Disagree; 3 = Neutral (neither agree nor disagree); 4 = Agree; 5 = Strongly Agree
Neighborhood safety and crime for parents	I feel safe walking in my neighborhood, day or night.	1 = Strongly Disagree; 2 = Disagree; 3 = Neutral (neither agree nor disagree); 4 = Agree; 5 = Strongly Agree
	Violence is not a problem in my neighborhood.	
	My neighborhood is safe from crime.	
